# Can Emergency Physicians Perform Carotid Artery Point-of-Care Ultrasound to Detect Stenosis in Patients with TIA and Stroke? A Pilot Study

**DOI:** 10.5811/westjem.2020.2.45137

**Published:** 2020-04-13

**Authors:** Robert Suttie, Michael Y. Woo, Lily Park, Marie-Joe Nemnom, Grant Stotts, Jeffrey J. Perry

**Affiliations:** * University of Ottawa, Department of Emergency Medicine, Ontario, Canada; † The Ottawa Hospital Research Institute, Ontario, Canada; ‡ University of Ottawa, Department of Medicine, Division of Neurology, Ontario, Canada

## Abstract

**Introduction:**

Patients with severe, symptomatic carotid stenosis can have their subsequent stroke risk reduced by surgical intervention if performed soon after a transient ischemic attack (TIA) or stroke. Patients presenting to an emergency department (ED) without computed tomography angiography (CTA) with TIA/stroke, may require transfer to another hospital for imaging to rule out carotid artery stenosis. The objective of this study was to determine the test characteristics of carotid artery point-of-care ultrasound (POCUS) in detecting greater than 50% stenosis in patients presenting with TIA/stroke.

**Methods:**

We conducted a prospective cohort study on a convenience sample of adult patients presenting to a comprehensive stroke centre with TIA or stroke between June–October 2017. Carotid POCUS was performed. Primary outcome measure, stenosis ≥ 50%, was determined by the final radiology report of CTA. A blinded POCUS expert separately reviewed the archived carotid POCUS scans. We calculated sensitivity and specificity for stenosis ≥ 50%.

**Results:**

We conducted POCUS on 75 patients, of which 70 were included in our analyses. Of those 70, 14.3% were diagnosed with greater than 50% stenosis. Carotid POCUS performed as follows: sensitivity 70.0% (95% confidence interval [CI], 34.8%–93.3%); specificity 86.7% (95% CI, 75.4%–94.1%); positive likelihood ratio (LR +) 5.3 (95% CI, 1.2–9.3); negative likelihood ratio (LR−) 0.4 (95% CI, 0.0–0.7). The inter-rater reliability between POCUS performer interpretation and expert interpretation had moderate agreement (k = 0.68). Scans took a mean 6.2 ± 2.2 minutes to complete.

**Conclusion:**

Carotid POCUS has low to moderate association with CTA for detection of carotid artery stenosis ≥ 50%. Further research and investigation is needed prior to widespread use of carotid POCUS in patients with acute cerebral ischemia. Additionally, external validity is likely affected by availability of training, maintenance of competency, and experience in more rural centres.

## INTRODUCTION

Stroke and transient ischemic attack (TIA) are relatively common reasons for emergency department (ED) visits. While TIA patients, by definition, fully recover, up to 23% will go on to have a subsequent stroke with 92% occurring in the following seven days.^1^–^3^ Carotid artery stenosis is a well-established etiology of TIA and stroke.^4^ Secondary stroke and death risk can be significantly reduced with carotid revascularization. This intervention is most beneficial for patients with 70–99% stenosis, with maximal benefit if completed within 14 days of the ischemic event and no further benefit after three months.^1^–^3^,^5^ There is also some benefit for patients with moderate stenosis (50–69%); however, the benefit is only present if surgery is performed within two weeks of the cerebral ischemic event.^1^–^3^,^5^

While the benefit of carotid revascularization is optimal when performed quickly, this is not occurring. Three Canadian studies have found that median delay to surgery from symptom onset varies from 25 to 79 days.^2^,^5^,^6^ Similar trends are observed in Europe with median times to surgery ranging from 53 to 82 days.^7^–^10^ Furthermore, studies have found that inpatients undergo carotid endarterectomy (CEA) more quickly with 54% receiving CEA within two weeks vs 20% compared to outpatients.^6^ The longest delay in care in one study was found to occur between symptom onset and vascular referral, which included delays to obtaining imaging given wait times for outpatient studies.^5^ In a second study, time to carotid imaging was the major factor determining time to revascularization.^10^ Having immediate access to comprehensive imaging may reduce delays to intervention. 5,10 This point is further emphasized in the recent Canadian Stroke Best Practice Guidelines from 2018.^11^

Computed tomography angiography (CTA) has long been the gold standard for the detection of intravascular stenosis of large arteries in the head and neck. However, to best serve ED patients it requires 24/7 access to a CT scanner as well as technologists and radiologists capable of accurately reading the scans. Unfortunately, only about 15% of Canadian rural EDs have access to a CT scanner 24/7.^12^ In the USA, similar trends are observed with less access to CT in smaller, rural hospitals.^42^ Another tool available is carotid duplex ultrasonography (DUS) with sensitivity for stenosis ≥ 50% of 96–98% and specificities of 83–88%.^22^, ^23^ However, DUS is quite complicated; it is usually performed by a dedicated ultrasound technician and requires extensive training. Additionally, DUS is often not available acutely or after hours.

Point-of-care ultrasonography (POCUS) is available in many EDs across the US and Canada and could be an adjunct to CT/DUS in hospitals with limited access.^13^,^41^ In addition to clinical information, risk-stratification of patients in the ED using POCUS could aid emergency physicians (EP) in their decision as to whether same day CTA is required. This would be particularly valuable to physicians in rural and community hospitals as this tool would help to efficiently determine who should be transferred to a tertiary care center for definitive imaging with CTA. Thus, the goal of this pilot study was to determine whether it is feasible for POCUS to detect carotid stenosis in patients with symptomatic TIA or stroke when compared with CTA.

## METHODS

### Study Design and Time Period, Study Setting and Population

We prospectively enrolled a convenience sample of ED patients seen at a tertiary care, comprehensive stroke center for either stroke or TIA from June to October 2017. Patients were recruited by the study team. Eligible patients had a diagnosis of stroke or TIA. Patients were excluded if they did not receive a CTA or if the POCUS was deemed indeterminate, which meant that the interpretation could not be deemed positive or negative. We obtained 68 final diagnoses and outcomes from chart review six months following the index ED visit. This consisted of an informal review of ED neurology consultations or outpatient Stroke Prevention Clinic documentation.

**Population Health Research Capsule**

What do we already know about this issue?

In some patients with carotid stenosis, stroke risk is reduced if surgery is performed quickly. Definitive imaging modalities aren’t always available.

What was the research question?

How well can carotid point-of-care ultrasound (POCUS) detect ≥ 50% stenosis in transient ischemic attack (TIA)/stroke patients?

What was the major finding of the study?

Compared to computed tomography angiography, carotid POCUS demonstrated a sensitivity of 70.0% with a specificity of 86.7%.

How does this improve population health?

For TIA/stroke patients in rural settings, carotid POCUS is a potential tool to expedite risk-stratification and transfer.

POCUS exams were completed in the ED during the index visit. A POCUS scanning protocol for the carotid artery was developed after a review of the literature and expert opinion. Standard B-mode and colour Doppler images were obtained for each patient following this protocol. The protocol did not include spectral Doppler or velocity calculations. The scanning protocol is included in Appendix 1.

**Figure 1:**
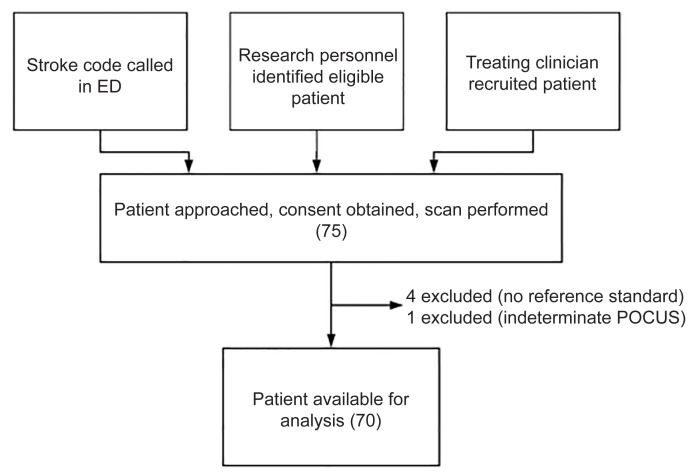
Recruitment flow chart for a study of the potential use of point-of-care ultrasound in patients presenting with stroke symptoms at rural hospitals. *ED*, emergency department; *POCUS*, point-of-care ultrasound.

Patients were recruited in a convenience fashion. During dedicated scanning shifts Monday to Friday from 9 am to 5 pm, a medical student recruited all stroke codes and patients evaluated for TIA/stroke when paged by the treating clinician. Additional patients were recruited by an emergency medicine (EM) resident during off-hours in the same fashion. Finally, a minority of patients were recruited by the treating clinician if they had been trained to complete the protocol (Figure 1).

### Intervention

Patients were scanned in the supine position using a high-frequency linear transducer Z-One Ultra (5–10 megahertz [MHz]) (Zonare, Mountain View, CA). Scans included the entire course of the common carotid and extracranial internal carotid artery (ICA) (Appendix 2, Images 1 and 2). An example of severe carotid stenosis is shown in Appendix 2, Images 3 and 4.

POCUS operators included a trained medical student, an EM resident and credentialed EPs at The Ottawa Hospital. Given there is no gold standard protocol available EPs were able to recruit after a one-hour didactic session with no hands-on practice. Most recruiting EPs were fellowship-trained in POCUS. The recruiting resident was responsible for developing the protocol and thus was responsible for leading the session. Training for the student consisted of two hours of didactic teaching and a four-hour supervised scanning shift, initially learning the basics of ultrasound and then the protocol, with credentialed POCUS EPs. The student completed 10 determinate carotid scans prior to recruitment. All images were archived for review by a blinded POCUS expert.

### Outcome Measures

The primary outcome of interest was carotid stenosis ≥50%. We chose ≥50% as opposed to ≥70% to ensure more conservative test characteristics. Degree of stenosis on POCUS was determined using an “eyeball” estimation. We used the final CTA report to ascertain the presence of stenosis and the percentage of stenosis if present. Radiologists were unaware of this study and blinded to the POCUS assessment. CTA was chosen as the reference standard as opposed to duplex ultrasound not because it is more accurate but because it is the current standard of care in the ED for these patients. Furthermore, our goal was to compare POCUS with the most widely used imaging practice. The secondary outcome was time required to complete a POCUS, which was generated from the image clips where the times of first and last scan were listed.

### Data Analyses

Data were collected from POCUS results and CTA for each patient, which was then compiled into a Microsoft Excel document (Microsoft Corporation, Redmond, WA) and imported into Statistical Analysis System (SAS Institute, Cary, NC) for analysis. We calculated sensitivity, specificity, predictive values, and likelihood ratios including 95% confidence intervals (CI). A kappa statistic described the inter-rater reliability between initial POCUS assessment and subsequent blinded expert review of the archived POCUS images.

## RESULTS

A total of 75 patients were scanned with 70 available for analysis (Table 1). Of the five patients excluded from the final analysis, four were excluded as they did not receive a CTA at the discretion of the treating physician because the etiology of their symptoms was not stroke/TIA and one was excluded for an indeterminate POCUS. Of the included patients, the average age was 70.4 years; 40 (57.1%) patients were male, 33 (47.1%) were diagnosed with stroke, 20 (28.6%) diagnosed with TIA, and 17 (24.3%) with a stroke mimic, including migraine, seizure, orthostatic hypotension, or encephalopathy. The majority of patients presented with mild deficits with 64 (91.4%) patients scoring a 6 or below on the National Institutes of Health Stroke Scale (NIHSS). Most patients had motor and sensory symptoms, with 27 patients (38.6%) presenting with language symptoms only and six (8.6%) presenting with ataxia only. Almost half of the cases (33 [47.1%] patients) were a stroke code activated by the EP.

At The Ottawa Hospital, a stroke code is called for expedited neurology assessment if the patient scores NIHSS 4 or more and has no contraindications to thrombolysis. Forty patients (57.1%) were discharged home. Of these patients, nine were diagnosed with stroke, 16 with TIA, and 12 with a stroke mimic. Ten patients had stenosis 50% or more diagnosed on CTA. Five patients were 50–69%, three patients were 70–99%, and two were 100% occluded. None of the patients with carotid stenosis between 50–69% or 100% had carotid revascularization within two weeks of the index event while all three cases between 70–99% received carotid revascularization. None of the patients with false negative POCUS went to surgery.

Carotid POCUS was poor to moderate in comparison to CTA (Table 2). Test characteristics were as follows: sensitivity 70.0% (95% CI, 34.8%–93.3%); specificity 86.7% (95% CI, 75.4%–94.1%); positive likelihood ratio (LR +) 5.3 (95% CI, 1.2–9.3); and LR – 0.4 (95% CI, 0.0–0.7). We calculated a kappa value of 0.68 (95% CI, 0.46–0.90) to compare initial interpretation and expert POCUS interpretation of scans, describing moderate agreement. There were three false negatives on POCUS: one with 100% stenosis and two with exactly 50% stenosis with all three occurring in the internal carotid artery.

Performance of carotid POCUS took a mean time of 6.2 **±** 2.2 minutes to complete. Maximum time was 12 minutes and minimum time was two minutes. This did not include the time to set up the ultrasound machine or any potential pre-scanning to the first saved image.

## DISCUSSION

Based on these results in patients with symptomatic TIA or stroke, carotid POCUS has a low to moderate association with CTA for the detection of 50% or greater carotid stenosis. Our patient cohort had a 14.3% prevalence of ≥ 50% stenosis, or 10 total patients. This seems to agree with existing literature that reports carotid artery stenosis as a cause of ischemic TIA and stroke in 10–15% of patients.^15^–^17^ Only three of 10 patients received intervention with either CEA or carotid stenting. Of the seven remaining patients all had moderate stenosis of 50–69% or a 100% occluded carotid; none of these were operated on at time of publication. Two had 100% chronically occluded arteries for which carotid revascularization was contraindicated.^43^ A third patient was eventually diagnosed with a seizure as the cause of his neurological symptoms, and thus the neurology team felt the carotid stenosis was incidental. One patient declined follow up due to ongoing health issues and thus was never assessed for surgery. Finally, three patients, all with exactly 50% carotid stenosis, were not intervened upon at the discretion of the treating inpatient team in keeping with most guidelines.^43^ All three patients with carotid stenosis 70–99% received intervention within two weeks.

The test characteristics only demonstrated low to moderate sensitivity and specificity, which was due to the three false negatives. An investigation of these three cases was performed. Two patients were found to have 50% stenosis of the left ICA with the other found to have 100% stenosis of the ICA just distal to its origin. All three of these patients were admitted. None of the three patients went on to have carotid revascularization at any point in their hospital stay or within three months following their hospitalization as it is not indicated for this patient group.^43^

One of the patients with a 50% stenosis was thought to have a free-floating thrombus after a small luminal defect was found. This patient had a unique ED course in that symptoms fluctuated in a crescendo TIA pattern and was seen by the neurology team twice in the same ED visit. On the first assessment, the CTA preliminary report did not identify the luminal defect and the patient was set to be discharged home. Neurology was called back to assess the patient due to recurrence of symptoms and on reassessment of the CTA, the defect was found. After anticoagulation, repeat CTA five days later did not demonstrate the defect. This was a high-risk, unstable TIA patient and in that clinical context a negative ultrasound should not change the patient course.

The second false negative patient was found to have a 100% stenosis, which was not intervened upon as there is no indication for revascularization of chronic occlusions.^38^, ^43^ This was likely missed due to significant amounts of plaque calcification that the US waves are unable to penetrate. The final false negative experienced a cerebellar event and, therefore, the carotid stenosis was deemed incidental and asymptomatic and thus not appropriate for carotid revascularization.

This is the first study describing the potential use of a novel carotid POCUS protocol to determine >50% carotid stenosis in patients presenting with TIA or stroke. When comparing comprehensive carotid duplex scanning in radiology departments, which includes velocity calculations to either angiography or CTA, sensitivity for carotid duplex scan for stenosis ≥50% has been previously found to be 96–98%, with specificities of 83–88%.^22^,^23^ While the specificity of POCUS is similar to duplex scanning, based on our study the sensitivity of POCUS appears to be quite a bit lower. It should also be stressed that this tool would only apply to TIA and non-disabling stroke in a clinical context where rapid outpatient imaging is available. With normal carotid imaging, patients presenting with stroke with disabling deficits should still be screened with CTA to determine candidacy for endovascular thrombectomy.

Additionally, images were generated by both novice and experienced POCUS sonographers after a short training period. While the results were modest, this speaks to the potential generalizability of carotid POCUS to this patient population. However, to simplify the protocol we elected to omit Doppler flow velocities and objective diameter measurements in lieu of an “eyeball” method. This may have had an effect on the overall validity of the test.

## LIMITATIONS

The small sample size and convenience sample are limitations in that there was likely some selection bias. Additionally, the study was underpowered secondary to the sample size and thus limits the reliability of the test characteristics. While the sensitivity generated was 70% it may have been as low as 34.8% due to wide confidence intervals. The results, however, do demonstrate a similar rate of stenosis compared with other reported studies. Almost half of the patients recruited were stroke codes that could limit the generalizability to a TIA population. A proportion of the patients recruited were not diagnosed with stroke or TIA by a stroke neurologist in lieu of a common stroke mimic, but this may be found in other patients presenting to the ED with TIA/stroke like symptoms. We also had one scan that was indeterminate due to inability to visualize the entire carotid. This scan was excluded as this is how POCUS is used clinically. With any indeterminate scan the clinician should disregard the results and act based on the clinical information they have; these patients should be transferred for more comprehensive imaging.

The event rate was also low and, therefore, further study is required prior to carotid POCUS being used to rule out 50% or greater carotid stenosis. It is also important to note that the inter-rater reliability of interpreting the scans was moderate, which may limit its external application. Finally, if further research is positive, this tool may be limited by its real-life applicability. Despite the generalizability, rural emergency physicians may not feel confident in a high-risk diagnosis using their own POCUS. This would have to be a target for educational interventions and continuing training/credentialing. Additionally, in a,higher resource medical system outpatient access to carotid imaging may not be delayed so as to affect surgical timeline. This likely limits the applicability of the scan to lower resource settings.

Because POCUS is available in most EDs.^41^ it is a potential tool for a common presentation in TIA/stroke patients, which includes risk-stratification and triaging transfers of these patients as well as rapid identification of stroke etiology. It likely would play less of a role in a tertiary care center with 24-hour access to CTA. Unfortunately, these sites are less likely to have access to POCUS and operators are likely less experienced, which would limit the generalizability of the tool. Additionally, there are other reasons for performing a CTA other than to determine carotid stenosis including identification of proximal thrombi amenable to thrombectomy.^39^

Additionally, CTA can predict patients at risk of clinical deterioration.^40^ Of course, because POCUS can only interrogate extracranial vessels, despite a normal scan these clinical questions would still require CTA or transfer to tertiary care. Unfortunately, due to the underpowering of our study the confidence intervals are quite wide and the test characteristics of the study may not be reliable. While our study demonstrates an intriguing new use of POCUS, it will require more study with more robust methodology until applied clinically.

## CONCLUSION

In our study, carotid POCUS had a low to moderate association with CTA for the detection of carotid stenosis greater than or equal to 50%. Further research and investigation is needed prior to widespread use of carotid POCUS in patients with acute cerebral ischemia.

## Supplementary Information





## Figures and Tables

**Table 1:** Characteristics of the patients available for analysis.

Characteristics	Number of patients (%)
Mean age, years (SD)	70.4 (13.4)
Male, n (%)	40 (57.1)
Co-morbidities, n (%)	
Previous TIA/stroke	21 (30)
Atrial fibrillation	14 (20)
Diabetes including type 1 & 2	16 (23)
Dyslipidemia	26 (37)
Hypertension	32 (45)
Peripheral vascular disease	7 (10)
Mechanical heart valve	1 (1.4)
Current medications, n (%)	
Anti-platelet*	33 (47)
Warfarin	4 (5.7)
DOAC	9 (13)
Statin	31 (44)
Eventual final diagnosis	
Stroke, n (%)	33 (47.1)
TIA, n (%)	20 (28.6)
Other	17 (24.3)
NIHSS on presentation, n (%)	
0	26 (37.1)
1–4	34 (48.6)
5–15	7 (10.0)
16–20	0 (0.0)
21–42	1 (1.4)
Unable to record†	1 (1.4)
Symptoms (may have more than one), n (%)	
Motor	41 (58.6)
Sensory	40 (57.1)
Language	27 (38.6)
Ataxia	6 (8.6)
Other‡	3 (4.2)
Time from symptom onset to ED presentation, minutes‡	
Range	30–7200
Mean	767
Mean excluding outliers§	142
Stroke code n, (%)	33 (47.1)
CTA, n (%)	
Under 50%	60 (85.7)
50% or over	10 (14.3)
Severe stenosis (over ≥ 50%) by NIHSS, n (% of cohort)	
0	3 (11.5)
1–4	6 (17.6)
5–15	0 (0)
16–20	0 (0)
21–42	1 (100)
Admitted, n (%)	30 (42.9)
Carotid revascularization within 2 weeks of index event of those with carotid stenosis, n (%)	3 (30.0)

*SD*, standard deviation; *TIA*, transient ischemic attack; *NIHSS*, National Institutes of Health Stroke Scale; *CT*, computed tomography; *DOAC*, direct oral anticoagulant; *PVD*, peripheral vascular disease; *POCUS*, point of care ultrasound; *ED*, emergency department.

* Includes ASA, clopidogrel, dipyramidole either single or dual antiplatelet

† Patient intubated and sedated prior to arrival at tertiary care center.

‡ Excluded 7 patients (no documented time of onset) and 2 patients (onset occurred within ED).

§ Excludes patients presenting over 24 hours (n = 13).

**Table 2:** Two-by-two table of carotid point-of-care ultrasound vs computed tomography angiography for carotid stenosis.

Carotid stenosis (≥ 50%)				Total
Carotid POCUS		(+)	(−)	
	(+)	7	8	15
	(−)	3	52	55

Total		10	60	70

		SS = 0.70	SP = 0.867	

*POCUS*, point-of-care ultrasound; *PPV*, positive predictive value; *NPV*, negative predictive value; *SS*, sensitivity; *SP*, specificity.

## References

[1] Giles M, Rothwell P (2007). Risk of stroke early after transient ischaemic attack: a systematic review and meta-analysis. Lancet Neurol.

[2] Gladstone D, Oh J, Fang J (2009). Urgency of carotid endarterectomy for secondary stroke prevention: results from the Registry of the Canadian Stroke Network. Stroke.

[3] Qureshi M, Davies A (2016). Carotid intervention following transient ischaemic attack: What are we waiting for?. Vascular.

[4] Chambers B, Norris J (1986). Outcome in patients with asymptomatic neck bruits. N Engl J Med.

[5] Jetty P, Husereau D, Kubelik D (2012). Wait times among patients with symptomatic carotid artery stenosis requiring carotid endarterectomy for stroke prevention. J Vasc Surg.

[6] Charbonneau P, Bonaventure PL, Drudi LM (2016). An institutional study of time delays for symptomatic carotid endarterectomy. J Vasc Surg.

[7] Johansson E, Wester P (2008). Delay from symptoms to carotid endarterectomy. J Intern Med.

[8] Dellagrammaticas D, Lewis S, Colam B (2007). Carotid endarterectomy in the UK: acceptable risks but unacceptable delays. Clin Med (Lond).

[9] den Hartog AG, Moll FL, van der Worp HB (2014). Delay to carotid endarterectomy in patients with symptomatic carotid artery stenosis. Eur J Vasc Endovasc Surg.

[10] Gocan S, Bourgoin A, Blacquiere D (2016). Fast-track systems improve timely carotid endarterectomy in stroke prevention outpatients. Can J Neurol Sci.

[11] Boulanger JM, Lindsay MP, Gubits G (2018). Canadian Stroke Best Practice Recommendations for Acute Stroke Management: Prehospital, Emergency Department, and Acute Inpatient Stroke Care, 6th Edition, Update 2018. Int J Stroke.

[12] Bergeron C, Fleet R, Tounkara F (2017). Lack of CT scanner in a rural emergency department increases inter-facility transfers: a pilot study. BMC Res Notes.

[13] Léger P, Fleet R, Giguère J (2015). A majority of rural emergency departments in the province of Quebec use point-of-care ultrasound: a cross-sectional survey. BMC Emerg Med.

[14] Adams H, Davis P, Leira E (1999). Baseline NIH Stroke Scale score strongly predicts outcome after stroke: a report of the Trial of Org 10172 in Acute Stroke Treatment (TOAST). Neurology.

[15] Hill M, Yiannakoulias N, Jeerakathil T (2004). The high risk of stroke immediately after transient ischemic attack: a population-based study. Neurology.

[16] Mohr J, Mast H (2011). Carotid artery disease. Stroke.

[17] Lal B, Adelman M, Indes J (2018). Carotid Artery Stenosis - BMJ Best Practice.

[18] Flaherty M, Kissela B, Khoury J (2012). Carotid artery stenosis as a cause of stroke. Neuroepidemiology.

[19] Al-Khaled M, Scheef B (2015). Symptomatic carotid stenosis and stroke risk in patients with transient ischemic attack according to the tissue-based definition. Int J Neurosci.

[20] Sheehan O, Kyne L, Kelly L (2010). Population-based study of ABCD2 score, carotid stenosis, and atrial fibrillation for Early stroke prediction after transient ischemic attack: the North Dublin TIA Study. Stroke.

[21] Tsivgoulis G, Spengos K, Manta P (2006). Validation of the ABCD score in identifying individuals at high early risk of stroke after a transient ischemic attack: a hospital-based case series study. Stroke.

[22] Henry J, Kiser D, Satiani B (2015). A critical evaluation of carotid duplex scanning in the diagnosis of significant carotid artery occlusive disease. Advances in Vascular Medicine.

[23] Jahromi A, Cinà C, Liu Y Sensitivity and specificity of color duplex ultrasound measurement in the estimation of internal carotid artery stenosis: A systematic review and meta-analysis. J Vasc Surg.

[24] Arger PH, De Bari Iyoob S (2015). Complete Guide To Vascular Ultrasound.

[25] Bhandari T, Socransky SJ (2014). Is B-mode ultrasound alone a sufficient screening tool for carotid stenosis? a pilot study. Crit Ultrasound J.

[26] Bluth E, Benson C, Ralls P (2008). Ultrasonography In Vascular Diseases: A Practical Approach.

[27] Carroll BA (1991). Carotid sonography. Radiology.

[28] Lee W (2013). General principles of carotid Doppler ultrasonography. Ultrasonography.

[29] Luengo-Fernandez R, Gray A, Rothwell P (2009). Effect of urgent treatment for transient ischaemic attack and minor stroke on disability and hospital costs (EXPRESS study): a prospective population-based sequential comparison. Lancet Neurol.

[30] McGahan J, Goldberg B (2008). Diagnostic Ultrasound.

[31] Myers K, Clough A (2014). Practical Vascular Ultrasound.

[32] Nicolaides A (2012). Ultrasound And Carotid Bifurcation Atherosclerosis.

[33] Polak J (2004). Peripheral Vascular Sonography.

[34] Rerkasem K, Rothwell P (2011). A systematic review of randomized controlled trials of carotid endarterectomy for symptomatic carotid stenosis. Stroke.

[35] Rothwell PM, Giles MF, Chandratheva E (2007). Effect of urgent treatment of transient ischaemic attack and minor stroke on early recurrent stroke (EXPRESS study): a prospective population-based sequential comparison. Lancet.

[36] Thomas K, Lewis N, Hill B (2015). Technical recommendations for the use of carotid duplex ultrasound for the assessment of extracranial blood flow. Am J Physiol Regul Integr Comp Physiol.

[37] Wardlaw J, Stevenson M, Chappell F (2009). Carotid artery imaging for secondary stroke prevention. Stroke.

[38] Ricotta J, AbuRahma A, Ascher E (2011). Updated Society for Vascular Surgery guidelines for management of extracranial carotid disease: Executive summary. J Vasc Surg.

[39] Nogueira R, Jadhav A, Haussen D (2018). Thrombectomy 6 to 24 hours after stroke with a mismatch between deficit and infarct. N Engl J Med.

[40] Coutts SB, Modi J, Patel SK (2012). What causes disability after transient ischemic attack and minor stroke? Results from the CT and MRI in the Triage of TIA and minor Cerebrovascular Events to Identify High Risk Patients (CATCH) Study. Stroke.

[41] Mengarelli M, Nepusz A, Kondrashova T (2018). A comparison of point-of-care ultrasonography use in rural versus urban emergency departments throughout Missouri. Mo Med.

[42] Ginde A, Foianini A, Renner D (2008). Availability and quality of computed tomography and magnetic resonance imaging equipment in U.S. emergency departments. Acad Emerg Med.

[43] Brott T, Halperin J, Abbara S (2011). Guideline on the management of patients with extracranial carotid and vertebral artery disease. J Am Coll Cardiol.

