# Supplementary material for: Can Emergency Physicians Perform Carotid Artery Point-of-Care Ultrasound to Detect Stenosis in Patients with TIA and Stroke? A Pilot Study
Source: West J Emerg Med. 2020 Apr 13;21(3):626–32. doi: 10.5811/westjem.2020.2.45137 (PMC7234698; doi:10.5811/westjem.2020.2.45137)
Supplement: Supplementary file 1 [file wjem-21-626-s001.docx]

**Appendix 1**

**Scanning Protocol ^[29, 30, 31, 32]^**

Protocol was developed from available ultrasonography literature and expert opinion including POCUS experts at the University of Toronto (Dr. J. Chenkin, Dr. S. Socransky, Dr. R. Simard) and a qualified vascular sonographer working at The Ottawa Hospital. Comprehensive carotid ultrasonography involves greyscale 2D images, colour and Doppler flow and velocity measurements to provide information regarding carotid stenosis. This is a technically challenging exam requiring significant training, experience, and time to perform. A limited protocol was developed after review of the literature and further discussion with sonographers and POCUS experts.

Patients were positioned supine for the duration of the examination. If they were unable to lie supine, they were positioned semi-recumbent at 45 degrees. The patient’s neck was slightly hyper-extended using a small pillow or rolled-up towel under the shoulders for support unless limited by pain or immobility of the cervical spine. The patient turned his or her head slightly away from the examiner, about 45 degrees from the midline, to enhance exposure.

A high-frequency linear transducer at 5-10 MHz was used with the ultrasound machine and vascular – carotid ultrasound preset. Scans began in the transverse plane with appropriate identification of the carotid artery (Figure 1). Key areas identified were the common carotid artery, carotid bulb and bifurcation, and ICA. The operator also scanned in the longitudinal plane. Confirmation of the ICA was done using both B-mode and colour mode. An initial sweep was conducted of the entire extracranial carotid as available, looking for obvious stenosis. If these were not encountered, transverse and longitudinal images were generated with colour over the vessel. The aim was to identify subtle or isoechoic stenosis that could potentially be missed on B-mode. This protocol was repeated on the opposite side.
