# Supplementary material for: Can Emergency Physicians Perform Carotid Artery Point-of-Care Ultrasound to Detect Stenosis in Patients with TIA and Stroke? A Pilot Study
Source: West J Emerg Med. 2020 Apr 13;21(3):626–32. doi: 10.5811/westjem.2020.2.45137 (PMC7234698; doi:10.5811/westjem.2020.2.45137)
Supplement: Supplementary file 2 [file wjem-21-626-s002.docx]

**Appendix 2**

| **Image 1.** Transverse image just above the clavicle demonstrating the common carotid artery (CCA) and internal jugular vein (IJV).. |
| --- |
| 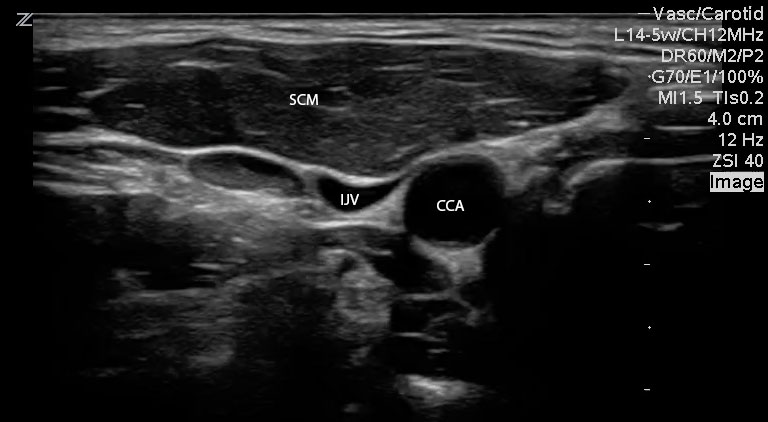 |
| CCA, common carotid artery; IJV, internal jugular vein,;SCM, sternocleidomastoid |

| **Image 2.** Transverse image of the internal carotid artery (ICA), external carotid artery (ECA) and internal jugular vein (IJV) |
| --- |
| 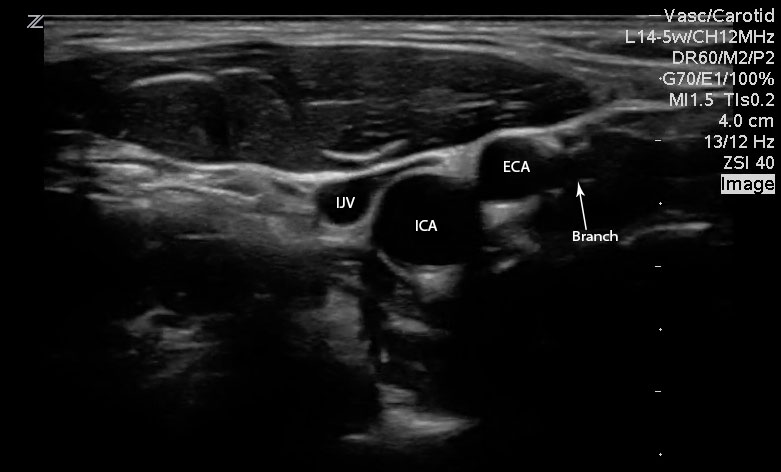 |
| IJV, internal jugular vein; SCM, sternocleidomastoid; ICA, internal carotid artery; ECA, external carotid artery. |

| **Image 3.** Longitudinal image of the internal carotid artery (ICA) with free-floating thrombus greater than 50% stenosis. |
| --- |
| 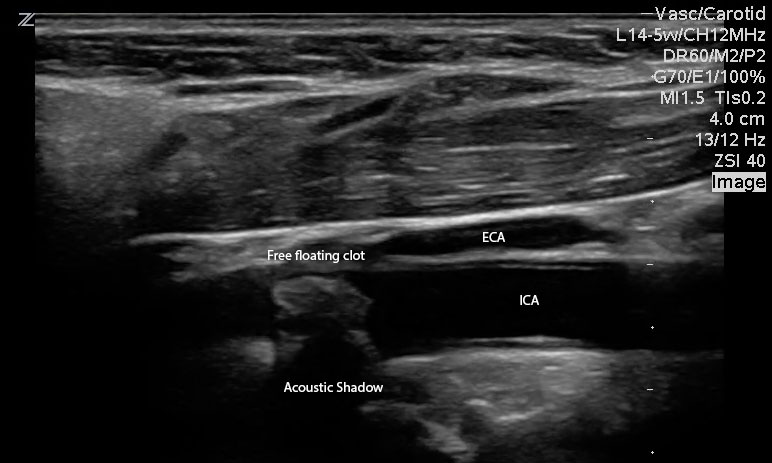 |
| ICA, internal carotid artery; ECA, external carotid artery. |

| **Image 4.** Longitudinal image of the internal carotid artery with colour Doppler demonstrating greater than 50% stenosis. |
| --- |
| 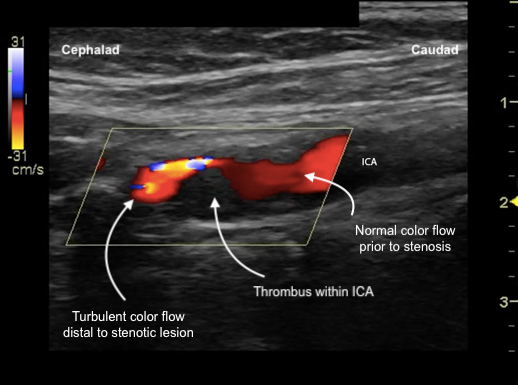 |
| ICA, internal carotid artery. Note the thrombus appears of similar echotexture compared to the blood within the vasculature. This highlights the importance of screening using colour as well as B-mode. |
